# Opportunity or Risk? Appraisal and Affect Mediate the Effect of Task Framing on Working Memory Performance in University Students

**DOI:** 10.3389/fpsyg.2021.615329

**Published:** 2021-09-01

**Authors:** Luxi Chen, Li Qu

**Affiliations:** ^1^Centre for Family and Population Research, National University of Singapore, Singapore, Singapore; ^2^School of Social Sciences, Nanyang Technological University, Singapore, Singapore

**Keywords:** working memory, appraisal, challenge, threat, affect, framing, gender

## Abstract

Working memory (WM) is crucial for reasoning, learning, decision-making and academic achievement. In diverse contexts, how a task is framed pertaining to its demands and consequences can influence participants' task performance by modifying their cognitive appraisals. However, less is known about the effect of task framing on WM performance and the mechanisms. This study examined whether opportunity- and risk-focused task framing would influence university students' WM performance by altering their cognitive appraisals and affective experiences. Ninety-seven university students were randomly assigned to one of the three framing conditions (Opportunity, Risk, vs. Null), and received instructions that differed in consequences (gain for top performers, loss for poor performers, vs. null), goals (approach, avoidance, vs. neutral), and feedback on personal competence (adequate, inadequate, vs. null). Challenge and threat appraisals, affect, and WM performance were measured before and after task framing. Results showed that opportunity-focused task framing improved students' WM performance, whilst risk-focused task framing increased threat appraisal and decreased positive affect, and that challenge appraisal was not altered in any condition. Female students were influenced by task framing to a greater extent than were male students. Mediation analysis revealed that the alteration of threat appraisal and the change in positive affect mediated the effect of task framing on WM performance. Findings highlight the important role of modifying cognitive appraisals and affective responses in optimizing cognitive performance.

## Introduction

University students often face important tasks such as examinations, public speaking and competitions, in which they are required to exert effort to perform well and achieve certain goals. These pressurized tasks were conceptualized as motivated performance situations, for their meaningful consequences, importance, and self-relevance (Seery, 2011). Working Memory (WM) is the capacity to store, retrieve and maintain activation of information in the execution of cognitive tasks, and it is involved in higher-order cognitive processes such as problem solving and reasoning (Engle, 2002; Conway et al., 2005). With central attention and complex cognitive processes (e.g., rehearsal, maintenance, updating and controlled search) as the key components, WM can facilitate students to perform better in various motivated performance tasks such as academic examinations, complex cognitive tasks, and social evaluation tasks. Hence, it is critical to explore how to optimize university students' WM performance in motivated performance contexts.

In diverse motivated performance situations (e.g., public speaking, motor tasks, sport competitions, and classroom examinations), task instructions that manipulated task demands and personal competence were found to influence participants' task performance by altering their cognitive appraisals (Lyons and Schneider, 2005; Moore et al., 2012; Jamieson et al., 2013, 2016; Beltzer et al., 2014). To be specific, when low task demand, high personal resources, or the potential benefits of stress were emphasized, participants were more likely to engage in challenge appraisal (i.e., interpretation of the task as a challenge or an opportunity), which further improved their task performance. Conversely, emphasizing high demand and/or required effort increased participants' threat appraisal (i.e., interpretation of the task as a threat or harm) which then debilitated their task performance. Induction of positive affect and alleviation of negative affect can also boost performance in exams (e.g., Beilock and Ramirez, 2011) and cognitive test performance such as WM (e.g., Yang et al., 2013). However, little is known about whether and how task framing concerning task demands and personal resources can influence WM performance.

### Task Framing and Cognitive Appraisals

According to the classic theory of emotion (Lazarus, 1991) and the transactional theory of stress (Lazarus and Folkman, 1984), an individual may interpret an encounter as “challenging” or “threatening” consciously and/or unconsciously at the initial stage, and this primary appraisal can be modified by secondary appraisal of personal resources and abilities to cope with the specific situation. People engaging in *challenge appraisal* tend to anticipate gain and growth from overcoming obstacles, whereas people engaging in *threat appraisal* tend to anticipate loss and harm. Furthermore, the biopsychosocial (BPS) model of challenge and threat (Blascovich and Tomaka, 1996; Blascovich et al., 2000; Blascovich, 2008a; Blascovich and Mendes, 2010) defined challenge and threat as two states that occur after one's evaluation of situational demands and personal resources, in goal-relevant performance contexts. Challenge is an approach-motivated state that occurs when people perceive adequate competence to meet situational demands, whilst threat is an avoidance-motivated state that occurs when evaluated situational demands exceed personal resources. As such, information about task demands (low vs. high), personal competence (adequate vs. inadequate), and achievement goals (approach vs. avoidance) may modify one's challenge and threat appraisals (for reviews, see Jones et al., 2009; Jamieson et al., 2018).

Consistent with the theoretical view, a series of experiments conducted by Moore et al. (2013a,b, 2014) showed that that instructing participants to focus on adequate personal resources to meet task demands successfully enhanced their challenge appraisal, whereas emphasizing high task demands and/or high required effort increased their threat appraisal. Moreover, information about the potential consequences conveyed by the experimenter can also alter participants' appraisals (Qu and Lim, 2016). In particular, highlighting the rewards for top performers prior to a motivated performance task engaged participants in a challenge state, whilst emphasizing the punishment for poor performers led them to a threat state (e.g., Moore et al., 2012, 2013b). Additionally, the contemporary achievement goal framework (Elliot and McGregor, 2001; Elliot, 2005) posited that an approach goal corresponds to higher levels of challenge appraisal, lower levels of threat appraisal, and more adaptive outcomes, compared to an avoidant goal in achievement contexts. Achievement goals explained half of the variance in threat and challenge appraisals in some studies (Elliot et al., 2000; McGregor and Elliot, 2002). Taken together, challenge appraisal may be boosted by opportunity-focused task framing that emphasizes an opportunity for gain, adequate personal competence, and an approach goal, whereas threat appraisal may be elicited by risk-focused task framing that emphasizes a risk of punishment, inadequate personal competence, and an avoidance goal.

### Appraisal, Affect, and Task Performance

The BPS model revealed the biological underpinnings of appraisal processes, and illustrated how challenge and threat determine affective responses, cognitive processes, as well as downstream performance and health outcomes. A challenge state activates challenge-type physiological responses (such as increased cardiac output and decreased peripheral resistance), increases positive affect like pride and excitement, promotes effective attention, and finally improves performance outcomes; contrariwise, people in a threat state are preoccupied with threat-type physiological responses (such as increased vascular resistance in anticipation of harms), experience negative affect like anxiety and shame, engage in ineffective attentional and cognitive processes, and eventually show worse task performance (Blascovich, 2008b; Blascovich and Mendes, 2010; Seery, 2011; Mendes and Park, 2014; Jamieson et al., 2016, 2018).

In supportive of the BPS model, numerous experiments have shown that the modification of challenge and threat appraisals resulted in the change in task performance (Jamieson et al., 2013, 2016). Threat appraisal elicited by task instructions has been associated with inferior performance, whereas challenge appraisal has been related to superior performance, in various motivated performance situations such as sports and motor tasks contexts, social evaluation tasks, and academic courses (e.g., Cerin et al., 2000; Seery et al., 2010; Moore et al., 2013b, 2014, 2015; Vine et al., 2013; Beltzer et al., 2014). The facilitating effect of a challenge state and the debilitating effect of a threat state on cognitive processes have also been found in some cognitive tasks such as the Stroop task (Turner et al., 2012), counting backward task (Tomaka et al., 1993; Schneider, 2004), and complex laboratory tasks (Gildea et al., 2007). Given that a challenge state can promote one's effective attention on task-relevant cues, whilst a threat state biases attention toward task-irrelevant or negative cues (Blascovich et al., 2004; Jones et al., 2009), it is plausible that WM performance can be improved by enhancing challenge appraisal and lowering threat appraisal, yet empirical investigation is still lacking.

Furthermore, modifying appraisals can alter one's affective experiences, which further contributes to the change in performance (e.g., Blascovich et al., 2004; Jones et al., 2009). The relation of challenge appraisal to positive affect and the relation of threat appraisal to negative affect have been well-established in the literature (for reviews, see Blascovich, 2008b; Jones et al., 2009; Seery, 2011; Jamieson et al., 2018). It has also been widely recognized that moderate positive affect can improve higher-order cognitive performance, such as creativity, problem solving, and decision making (see Isen, 2008, for a review). For instance, WM performance was positively associated with positive affect (Yang et al., 2013), and negatively associated with negative affect like subjective distress (Matthews and Campbell, 2010). With regard to whether affective responses mediate the association between task framing and performance, there have been mixed findings in the literature. Affect mediated the association between a mastery goal and WM performance (Linnenbrink et al., 1999), but it did not mediate the influence of task instructions that manipulated task demands and personal competence on motor task performance in some studies (e.g., Moore et al., 2013b).

Taken together, it is necessary to examine whether task instructions that manipulate task demands (low vs. high), personal competence (adequate vs. inadequate), consequences (opportunity for gain vs. risk of loss), and goals (approach vs. avoidance) influence university students' WM performance by altering their cognitive appraisals and affective responses.

### Gender Differences

Gender differences have been found in various psychological processes such as WM (e.g., Kaufman, 2007; Lynn and Irwing, 2008; Lejbak et al., 2011), cognitive appraisals and emotional states (e.g., Hankin and Abramson, 2001; Koch et al., 2007). In terms of cognitive task performance, men outperformed women in spatial and object WM, but not in verbal WM or digit span tasks (Kaufman, 2007; Lynn and Irwing, 2008; Lejbak et al., 2011). A meta-analysis conducted by Hill et al. (2014) revealed the neurophysiological basis of gender differences in WM and test performance. Moreover, men have shown better ability to cognitively control their emotions than women (Birditt and Fingerman, 2003; Koch et al., 2007). Females, by contrast, are more likely to engage in threat appraisal and avoidance-oriented behaviors (e.g., behavioral disengagement), which contribute to their greater vulnerability to negative emotional states than males (e.g., Hankin and Abramson, 2001; Chen and Qu, 2021). Hence, it is reasonable to expect that females may alter their appraisals and affective responses to a greater extent than males in a motivated performance task, and consequently their WM performance may be affected by task framing more greatly. This proposal, nonetheless, requires empirical examination.

### Present Study

To fill the gap in understanding the influences of task framing on WM performance and the possible mechanisms, this study experimentally investigated whether framing the WM task as an opportunity for gain (that an individual has adequate competence to approach) would improve WM performance, and whether framing the task as a risk of loss or cost (that a person has inadequate competence to avoid) would debilitate WM performance, by altering cognitive appraisals and affect. The second aim of the present study was to examine whether the effect of task framing on WM (if any) and the mechanisms would vary by gender.

We hypothesized that, firstly, the interaction effect between Framing Type (Opportunity, Risk, vs. Null) and Time (Pre- vs. Post-framing) would be found on all the variables, and specifically, (1a) participants in the Opportunity condition would increase challenge appraisal and positive affect, decrease threat appraisal and negative affect, and improve WM performance, from pre- to post-framing, whereas (1b) participants in the Risk condition would decrease challenge appraisal and positive affect, increase threat appraisal and negative affect, and decrease WM scores, from pre- to post-framing. Second, it was expected that (2a) the alteration of challenge and threat appraisals would mediate the influences of opportunity- and risk-focused task framing on the change in WM performance, and (2b) the alteration of positive and negative affect would act as the secondary mediator to mediate the association between the alteration of appraisals and the change in WM performance. Lastly, we expected to observe gender differences in the modification of scores from pre- to post-framing in all the major measures, and in particular, (3a) in the Risk condition, female students would experience a larger increase of threat appraisal and negative affect, as well as a larger decrease of challenge appraisal, positive affect, and WM scores, than male students, and (3b) in the Opportunity condition, female students would display a larger decrease of threat appraisal and negative affect, as well as a larger increase of challenge appraisal, positive affect, and WM scores, than male students.

## Materials and Methods

### Participants

Ninety-seven undergraduate students (60 females) with a mean age of 21.3 (*SD* = 1.78; range 18–27) years were recruited from the Research Pool of Nanyang Technological University in Singapore. They were randomly assigned to three framing conditions, namely, Opportunity (*N* = 31; 17 female), Risk (*N* = 31; 19 female), and Null (*N* = 35; 24 female). Each participant obtained three research course credits for compensation. The priori power analysis conducted on G^*^Power 3.1 (Faul et al., 2009) showed that the sample size was sufficient (66 were required) to detect the smallest meaningful effect size (*f* = 0.20) for repeated measures within-between mixed analysis of variance (ANOVA) (3 groups and 2 repetitions), with a minimum statistical power (1 – β) = 0.80, a significance level at 0.05, correlations among repeated measures *r* = 0.5, and non-sphericity correction ε = 1.

### Design and Procedure

The present study was an experimental, between- and within-subjects repeated measure mixed design. Framing Type (Opportunity, Risk, vs. Null) was the between-subjects factor, Time (Pre-framing vs. Post-framing) was the within-subjects factor, and the dependent variables included WM performance, challenge and threat appraisals, and positive and negative affect.

**Figure 1:**
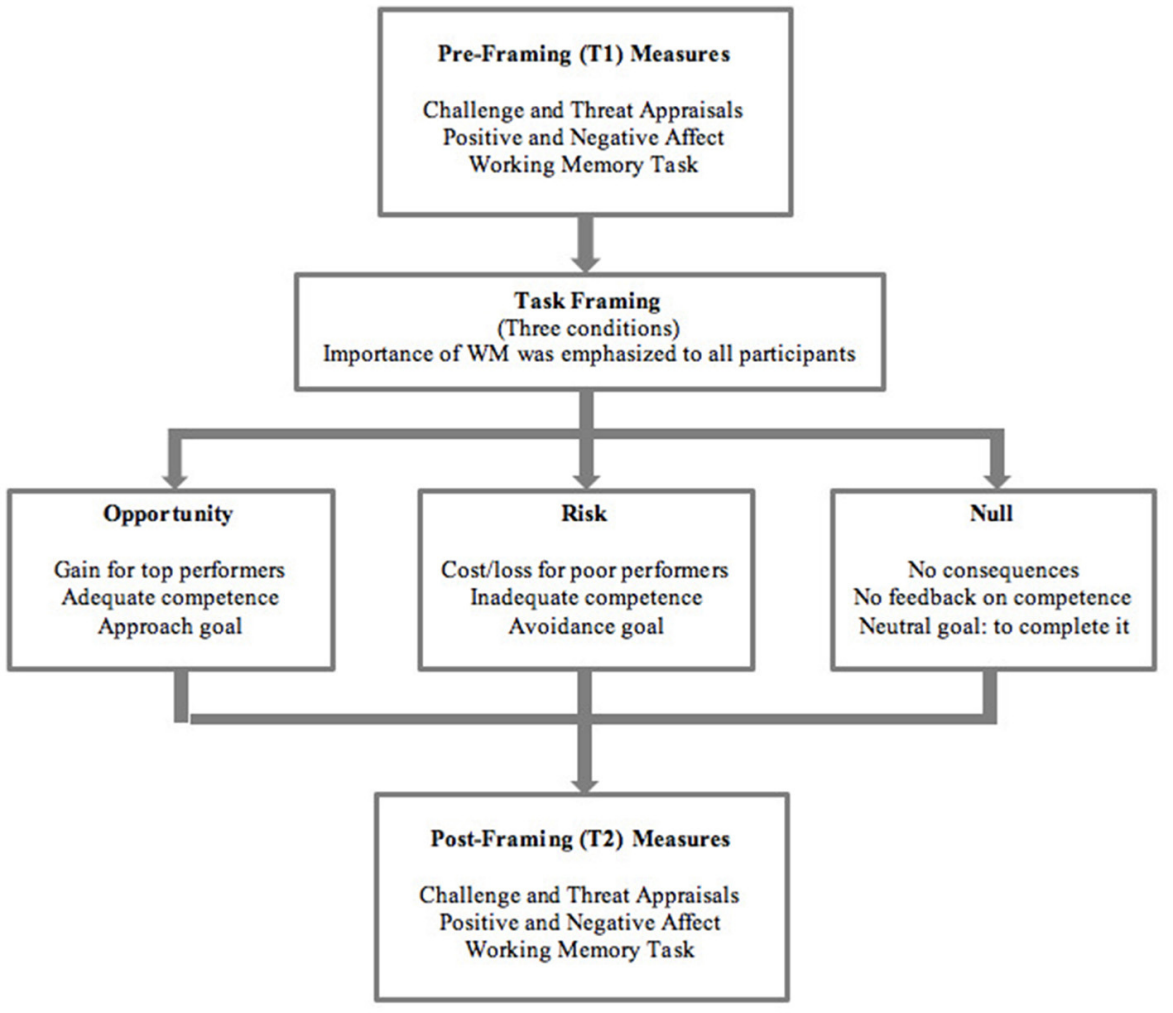
Overview of study procedure.

Each participant took part in the experiment individually in a quiet laboratory room. Participants indicated their informed consent and provided demographic information. The whole procedure (see Figure 1) lasted for <60 min. Participants were randomly assigned to Opportunity, Risk or Null condition. Before task framing (T1), all participants were required to complete the baseline WM task without receiving any information pertaining to its nature and consequences. Prior to the baseline task, participants reported their appraisals of the baseline task as well as their baseline positive and negative affect. After completing the baseline WM task, the experimenter revealed the nature of the task as an assessment of WM, with the importance of WM capacity in daily lives being emphasized to all participants. Participants were then informed that the previous task was a practice, and they were required to do the actual WM task again. Three groups received different instructions (i.e., opportunity-focused, risk-focused, and null) that differed in consequences, goals, and feedback on personal competence to meet task demands. After task framing (T2), participants reported their challenge and threat appraisals of the forthcoming WM task, positive and negative affect, and then completed the actual task. Debriefing was provided to all participants after the experiment.

### Measures

#### WM Task

The Forward Digit Span Task paradigm (Conway et al., 2005) was adopted to assess participants' WM capacity. Inquisit 4.0 was used to carry out the adaptive computerized WM task. Each participant was presented visually with a random series of digits (e.g., “9, 3, 5”) and required to repeat them immediately by entering the digits in the given order. If the participant repeated the numbers in one trial successfully, he or she would be given a longer number series in length by one digit. Number series for each trial was randomly selected from the pool of the particular length. The longest length of numbers they are successful in repeating (i.e., memory span) was indicative of WM performance in the current study. T1 and T2 WM scores were positively correlated with each other (*r* = 0.51, *p* < 0.001), indicating good reliability of the digit span task.

#### Challenge and Threat Appraisals

To measure challenge and threat appraisals of a specific task, four items were selected from the Chinese Making Sense of Adversity Scale (CMSAS; Pan et al., 2008) and modified to be task-specific. Two items measured challenge appraisal (“*the task is an opportunity for learning*” and “*the task is normal, and everyone has to face it here and there*”) and two items measured threat appraisal (“*the task may damage my self-esteem*” and “*I may lose a lot because of this task*”), on a 4-point scale ranging from 1 (*does not fit at all*) to 4 (*fits extremely well*). Both subscales exhibited acceptable internal consistencies in T1 and T2 (Cronbach's alphas were from 0.70 to 0.73). Within each measure, scores at both time points were highly correlated (challenge appraisal: *r* = 0.81, *p* < 0.001; threat appraisal: *r* = 0.57, *p* < 0.001), suggesting good reliabilities of both appraisal subscales.

#### Positive and Negative Affect

Participants rated their positive affect (4 items; “happy” “cheerful” “energetic” “inspired”) and negative affect (4 items; “Helpless” “Tense” “Nervous” “Upset”) from 1 (does not fit at all) to 4 (fits extremely well). The words were selected from the Positive and Negative Affect Schedule (PANAS; Watson et al., 1988). Both subscales displayed good internal consistencies at both time points (Cronbach's alphas were from 0.82 to 0.87). Within each measure, scores at both time points were highly correlated with each other (positive affect: *r* = 0.77, *p* < 0.001; negative affect: *r* = 0.61, *p* < 0.001).

#### Task Framing

Three framing conditions (Opportunity, Risk, vs. Null) were created by three types of task instructions that were different in consequence (opportunity for gain, risk of loss, vs. null), goal (approach, avoidance, vs. null) and self-competence (adequate, inadequate, vs. null). The task instructions were modified from previous experiments (e.g., Moore et al., 2012, 2013b) which succefully altered challenge and threat states and task performance in motor tasks. During task framing, the importance of the upcoming WM task was firstly emphasized to all participants, to foster task engagement. The Opportunity group was informed of the opportunity for gain (i.e., one additional research course credit as bonus for top performers, in addition to the three research credits as basic incentives), provided with positive feedback on adequate competence to meet the task demands, and encouraged to adopt an approach goal toward the reward. The Risk group was informed of the risk of loss or cost (i.e., poor performers would go through an additional interview conducted by their teaching assistant who would grade their quiz, essays, presentations and final exams in the current research module), provided with negative feedback on inadequate competence to meet the task demands, and encouraged to adopt an avoidance goal to avoid the punishment. The Null group did not receive extra information concerning the consequence, personal competence and goals, as a control. The instruction details are presented in Table 1.

**Table 1:** Instructions in three task framing conditions.

**Task framing components**	**Opportunity**	**Risk**	**Null**
Task engagement	**Importance and self-relevance**: The task you completed just now was a working memory task. Working memory is a very important cognitive capacity in our daily life that can predict academic success. The previous task was just a practice, and now you are required to do the actual task. The upcoming task is basically the same as the previous one.		
Consequence of the task	**Opportunity for gain**: We are looking for top performers in working memory task. Those who rank the top 10% in the actual task will be given one more research course credit as bonus.	**Risk of loss**: We are looking for students who perform poorly in working memory task. Those who rank the bottom 10% in the actual task will be required to go through an additional interview by their course teaching assistant to figure out why they perform worse than others.	**Null**: We are investigating what factors can influence working memory.
Personal competence	**Adequate**: You did very well in the practice trials. According to your practice scores, I have great confidence that you can rank the top 10% in the actual task.	**Inadequate**: Other participants did very well in the practice trials. According to your practice scores, you may have to try harder in the actual task if you don't want to lie in the bottom 10%.	**Null**: You have completed the practice trials just now.
Goal of the task	**Approach**: Please try your best to get the bonus.	**Avoidance**: Please try to avoid the punishment.	**Neutral**: Please complete the actual task again.

### Analytical Strategy

First of all, we examined whether the baselines (i.e., pre-test scores) among three conditions were comparable, using a series of ANOVAs, with framing type as the group factor, and pre-framing measures as the dependent variables. If the baselines were all comparable, 3 (Framing Type: Opportunity, Risk, vs. Null) × 2 (Time: Pre- vs. Post-test) repeated measures ANOVAs would be deployed to test the effect of framing type on the change in WM performance, appraisals and affect from pre- to post-test. If any of the baselines was not comparable, 3 (Framing Type) × 2 (Time) repeated measures analyses of covariance (ANCOVAs) would be performed, controlling for the particular incomparable pre-framing score(s). Bonferroni adjustment was applied for multiple comparison to control for familywise errors (α = 0.017). When the interaction effect was significant on a particular variable, paired samples *t*-tests within each condition were conducted to test the change from pre- to post-test, with the significant level for alpha value being adjusted to.05/3 = 0.017.

Next, PROCESS Procedure (Hayes, 2017) in the SPSS 25.0 software was performed to examine the mediating roles of the alteration of appraisals and the modification of affect in the association between task framing and the change in WM performance.

Finally, gender differences were examined by a series of 2 (Gender: male vs. female) × 3 (Framing Type: Opportunity, Risk, vs. Null) ANOVAs, with the alteration of cognitive appraisals, affect, and WM scores from pre- to post-test as the dependent variables. If the interaction effect was significant, data in each framing condition would be split by gender, and paired samples *t*-tests would be then performed to test the change within each gender in each condition. If gender difference was found, gender (dummy coded as girl = 1, boy = 0) would be entered as the moderator to the mediation model.

## Results

### Preliminary Analysis

Descriptive statistics results and correlations of study measures are presented in Table 2. Among concurrent measures, challenge appraisal was positively associated with positive affect, whereas threat appraisal was positively associated with negative affect, at both time points. Concurrently measured challenge appraisal was not associated with threat appraisal, and positive affect was not related to negative affect, at either time point. Pre-framing (T1) WM capacity was not associated with any other measures, whilst post-framing (T2) WM performance was negatively associated with both T1 and T2 threat appraisal.

**Table 2:** Means, standard deviations, and bivariate correlations of main variables.

	**1. T1 challenge appraisal**	**2. T1 threat appraisal**	**3. T1 positive affect**	**4. T1 negative affect**	**5. T1 WM**	**6. T2 challenge appraisal**	**7. T2 threat appraisal**	**8. T2 positive affect**	**9. T2 negative affect**	**10. T2 WM**
1	–									
2	−0.01	–								
3	0.42^***^	−0.24^*^	–							
4	0.04	0.59^***^	0.006	–						
5	0.05	−0.13	−0.03	−0.05	–					
6	0.81^***^	0.009	0.39^***^	−0.04	0.007	–				
7	0.01	0.57^***^	−0.12	0.43^***^	−0.19^+^	0.07	–			
8	0.42^***^	−0.08	0.77^***^	0.04	0.18^+^	0.45^***^	−0.17	–		
9	0.09	0.27^**^	0.16	0.61^***^	−0.11	−0.003	0.40^***^	0.04	–	
10	−0.08	−0.25^*^	0.03	−0.11	0.51^***^	−0.17	−0.37^***^	0.05	−0.12	–
*M*	2.82	1.19	2.44	1.52	8.39	2.30	1.59	2.74	0.68	8.62
*SD*	0.65	0.39	0.85	0.66	1.14	0.93	0.63	1.25	0.41	1.23

*** *p < 0.001*;

** *p < 0.001*;

* *p < 0.05*;

+ *p < 0.01. WM, Working Memory*.

Means and standard deviations of study variables over two time points in three conditions are presented in Table 3. Baselines were comparable across conditions in all variables (*ps* > 0.10) but positive affect [*F*(2, 94) = 4.05, *p* = 0.02]. In particular, Opportunity and Risk groups reported comparable baseline positive affect (*p* > 0.10), but the Null group reported a significantly lower level of positive affect than the Risk group (*p* = 0.03) and a marginally lower level than the Opportunity group (*p* = 0.06). Thus, T1 positive affect would be entered as the covariate in all analyses in the current study.

**Table 3:** Means and standard deviations of main variables over two time points in three conditions.

	**Opportunity condition (** ***n*** **= 31)**		**Risk condition (** ***n*** **= 31)**		**Null condition (** ***n*** **= 35)**			
	**T1**	**T2**	**T1**	**T2**	**T1**	**T2**	**Min**	**Max**
WM	8.42 (1.09)	9.03^a^^**^ (1.30)	8.16 (0.90)	8.35 (1.25)	8.57 (1.36)	8.49 (1.07)	6	11
Challenge appraisal	2.81 (0.73)	2.69 (0.73)	2.90 (0.51)	2.89 (0.59)	2.76 (0.69)	2.64 (0.71)	1	4
Threat appraisal	1.15 (0.32)	1.10 (0.20)	1.16 (0.35)	1.39^a^^**^ (0.38)	1.24 (0.48)	1.26 (0.52)	1	4
Positive affect	2.60 (0.77)	2.46 (0.89)	2.64 (0.57)	2.35^b^^***^ (0.93)	2.12 (0.93)	2.13 (0.97)	1	4
Negative affect	1.39 (0.48)	1.39 (0.35)	1.55 (0.73)	1.74^a^^+^ (0.64)	1.60 (0.73)	1.63 (0.76)	1	4

*Means and Standard Deviations (in parentheses) are presented*.

a *Significantly higher than baseline*.

b *Significantly lower than baseline*.

*** *p < 0.001*;

** *p < 0.01*;

+ *p < 0.10*.

### Effects of Task Framing

#### WM Performance

**Figure 2:**
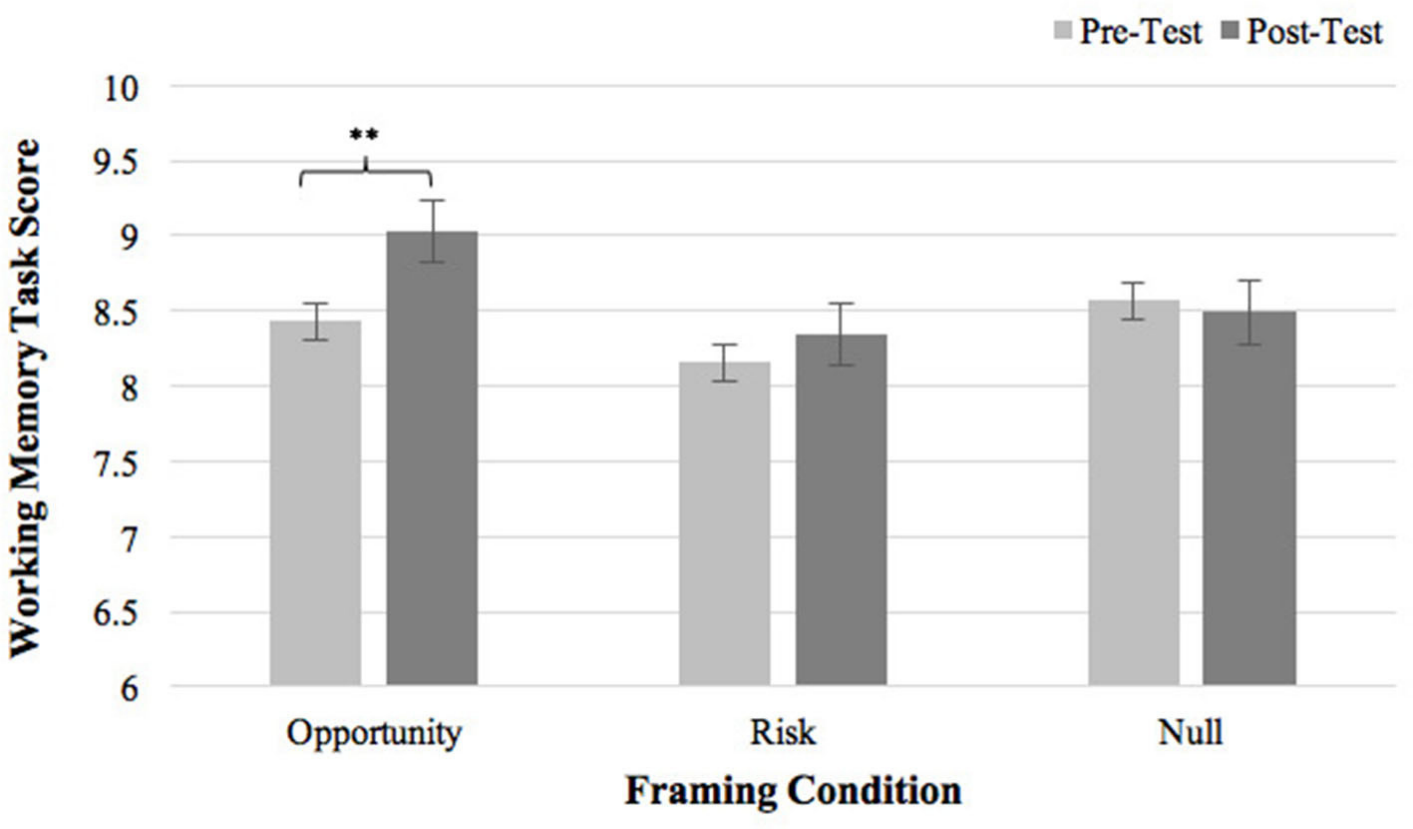
Pre- and post-framing WM scores in three framing conditions. Error bars represent standard errors. **p < 0.01.

ANCOVA yielded a marginally significant interaction effect of Framing Type × Time [*F*(2, 93) = 2.81, *p* = 0.065, η^2^ = 0.06] on WM performance. The main effect of Framing Type [*F*(2, 93) = 1.62, *p* = 0.20, η^2^ = 0.03] and the main effect of Time [*F*(1, 93) = 0.30, *p* = 0.59, η^2^ = 0.003] were both non-significant. As displayed in Figure 2, after receiving opportunity-focused task instruction, participants in the Opportunity condition significantly improved their WM scores compared to baseline (Δ*M* = 0.61, *t* = 2.98, *p* = 0.006). No change in WM performance from pre- to post-test was observed in the Risk condition (Δ*M* = 0.19, *t* = 0.81, *p* = 0.42) or the Null condition (Δ*M* = −0.086, *t* = −0.52, *p* = 0.61). Results suggested that opportunity-focused task instruction significantly improved WM performance.

#### Appraisals

To control for the potential influence of baseline WM performance on students' interpretation of the target WM task at T2, we entered T1 WM (in addition to T1 positive affect) as a covariate in repeated measures ANCOVAs on challenge and threat appraisals.

##### Challenge Appraisal

**Figure 3:**
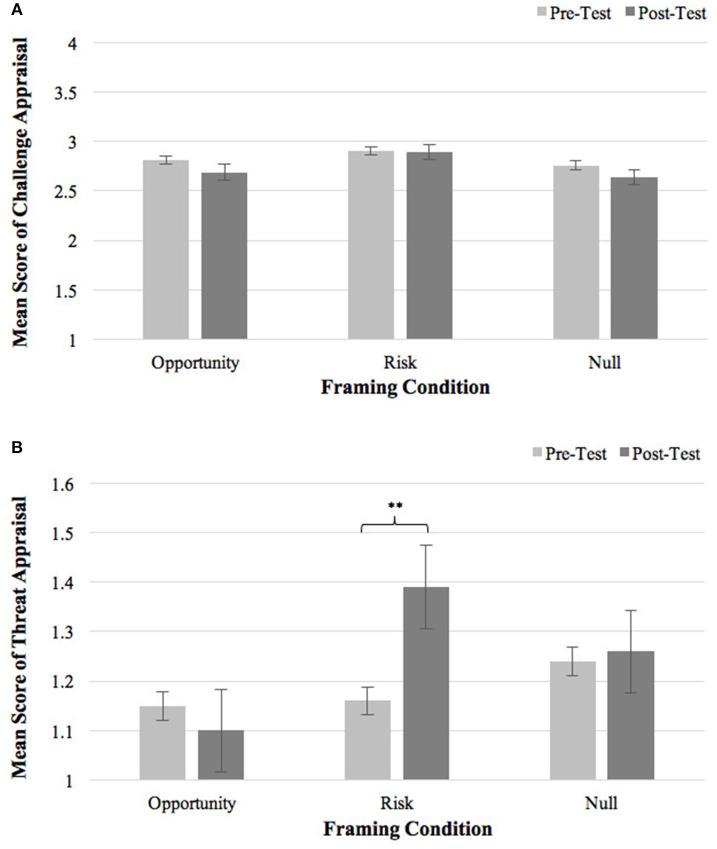
**(A)** Pre- and post-framing challenge appraisal in three framing conditions. **(B)** Pre- and post-framing threat appraisal in three framing conditions. Error bars represent standard errors. **p < 0.01.

None of the main effects and interaction effect was significant on challenge appraisal [Framing Type: *F*(2, 92) = 0.47, *p* = 0.63, η^2^ = 0.010; Time: *F*(1, 92) = 0.14, *p* = 0.71, η^2^ = 0.002; Framing Type x Time: *F*(2, 92) = 0.58, *p* = 0.61, η^2^ = 0.013], suggesting that task framing did not alter participants' challenge appraisal (see Figure 3A). Additional ANOVAs were conducted to examine the framing effect on the two items of challenge appraisal separately, but no significant effect was found (*ps* > 0.10).

##### Threat Appraisal

We observed a significant interaction effect of Framing Type × Time on threat appraisal [*F*(2, 92) = 4.56, *p* = 0.013, η^2^ = 0.090]. Neither the main effect of Framing Type [*F*(2, 92) = 1.39, *p* = 0.25, η^2^ = 0.029] nor the main effect of Time [*F*(1, 92) = 0.027, *p* = 0.87, η^2^ < 0.001] was significant. As shown in Figure 3B, after receiving risk-focused instruction, the Risk group significantly increased threat appraisal compared to baseline (Δ*M* = 0.23, *t* = 3.72, *p* = 0.001), and reported significantly higher post-framing threat appraisal than their counterparts in the Opportunity condition {*p* = 0.013, *95% CI* [−1.08, −0.097]}. No change in threat appraisal was found in the Opportunity condition (Δ*M* = −0.048, *t* = −0.77, *p* = 0.45) or the Null condition (Δ*M* = 0.014, *t* = 0.23, *p* = 0.82). Result indicated that risk-focused task instruction intensified threat appraisal.

#### Affect

##### Positive Affect

**Figure 4:**
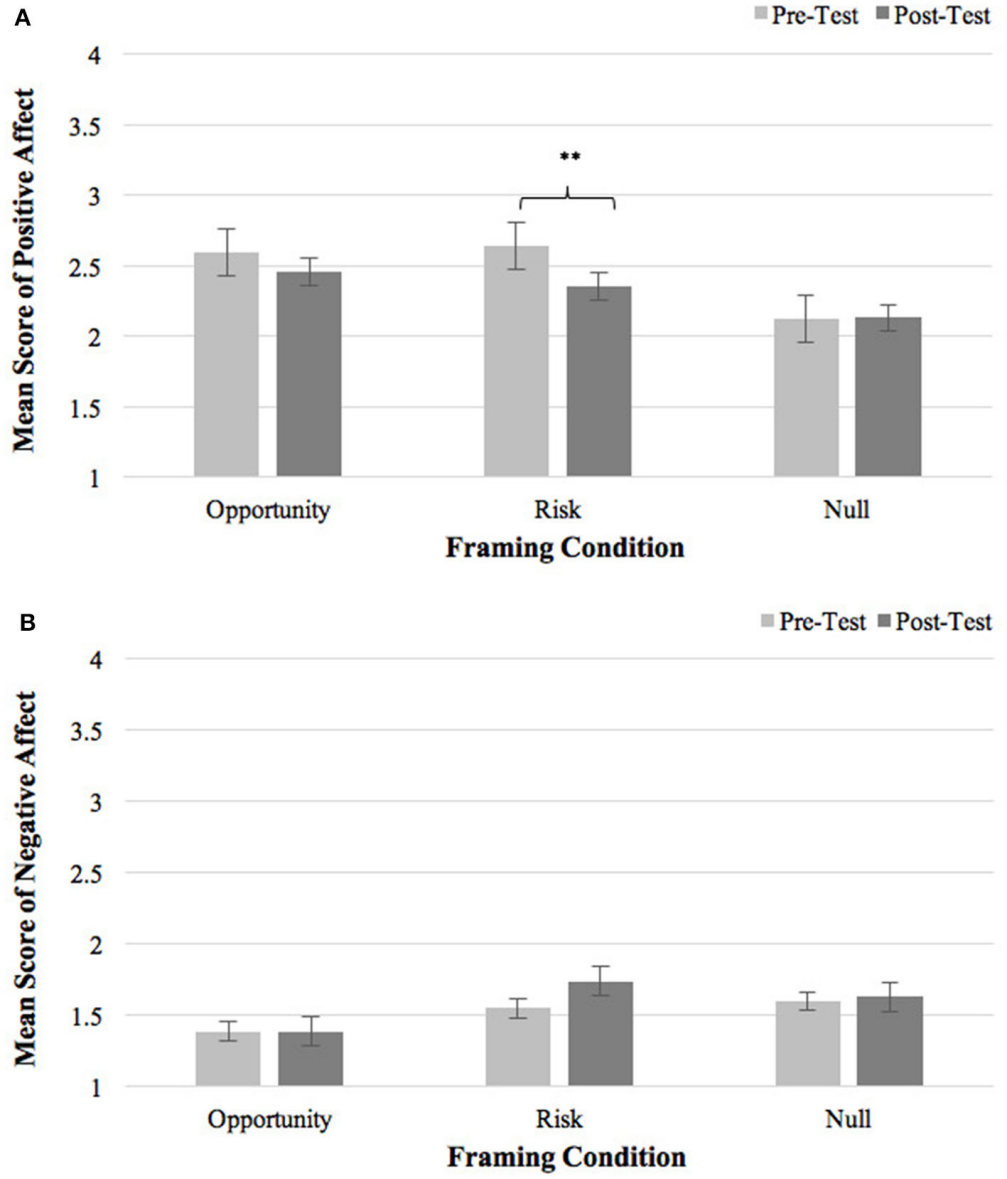
**(A)** Pre- and post-framing positive affect in three framing conditions. **(B)** Pre- and post-framing negative affect in three framing conditions. Error bars represent standard errors. **p < 0.01.

Only the main effect of Time [*F*(1,94) = 5.17, *p* = 0.03, η^2^ = 0.05] was found on positive affect, with a significant decrease from pre- to post-test [*t* = −2.16, *p* = 0.03]. The main effect of Framing Type [*F*(2, 94) = 2.43, *p* = 0.09, η^2^ = 0.06] and the interaction effect of Framing Type × Time [*F*(2, 94) = 1.96, *p* = 0.15, η^2^ = 0.04) were non-significant. As shown in Figure 4A, after receiving risk-focused instruction, the Risk group significantly reduced positive affect compared to the baseline (Δ*M* = −0.29, *t* = −3.15, *p* = 0.004). No change in positive affect was observed in the Opportunity condition (Δ*M* = −0.14, *t* = −1.11; *p* = 0.28) or the Null condition (Δ*M* = 0.007, *t* = 0.07; *p* = 0.94). Result suggested that risk-focused task instruction decreased participants' positive affect.

##### Negative Affect

None of the main effects and interaction effect was significant on negative affect [Framing Type: *F*(2, 93) = 1.25, *p* = 0.27, η^2^ = 0.013; Time: *F*(1, 93) = 1.04, *p* = 0.31, η^2^ = 0.011; Framing Type × Time: *F*(2, 93) = 0.88, *p* = 0.42, η^2^ = 0.018], indicating that task framing did not influence participants' negative affect (see Figure 4B).

In sum, opportunity-focused task instruction that emphasizes the potential gain for top performers, adequate personal competence and an approach goal significantly improved University students' WM performance. In contrast, risk-focused task instruction that emphasizes the loss for poor performers, inadequate personal competence and an avoidance goal intensified students' threat appraisal and reduced their positive affect.

### Mediating Roles of Appraisal and Affect

Based on the ANOVA results, we further investigated whether the effect of task framing on the change in WM performance was mediated by the alteration of threat appraisal and that of positive affect, in the whole sample. Task framing (3 conditions) was dummy coded as two variables, namely, Opportunity (1 = opportunity-focused instruction; 0 = risk-focused instruction or null instruction) and Risk (1 = risk-focused instruction; 0 = opportunity-focused instruction or null instruction). Opportunity and Risk acted as the predictors in the model of Opportunity and the model of Risk, respectively. In both models, the change in WM score from pre- to post-test was the outcome variable, and the alteration of threat appraisal and that of positive affect were entered as the mediators.

**Figure 5:**
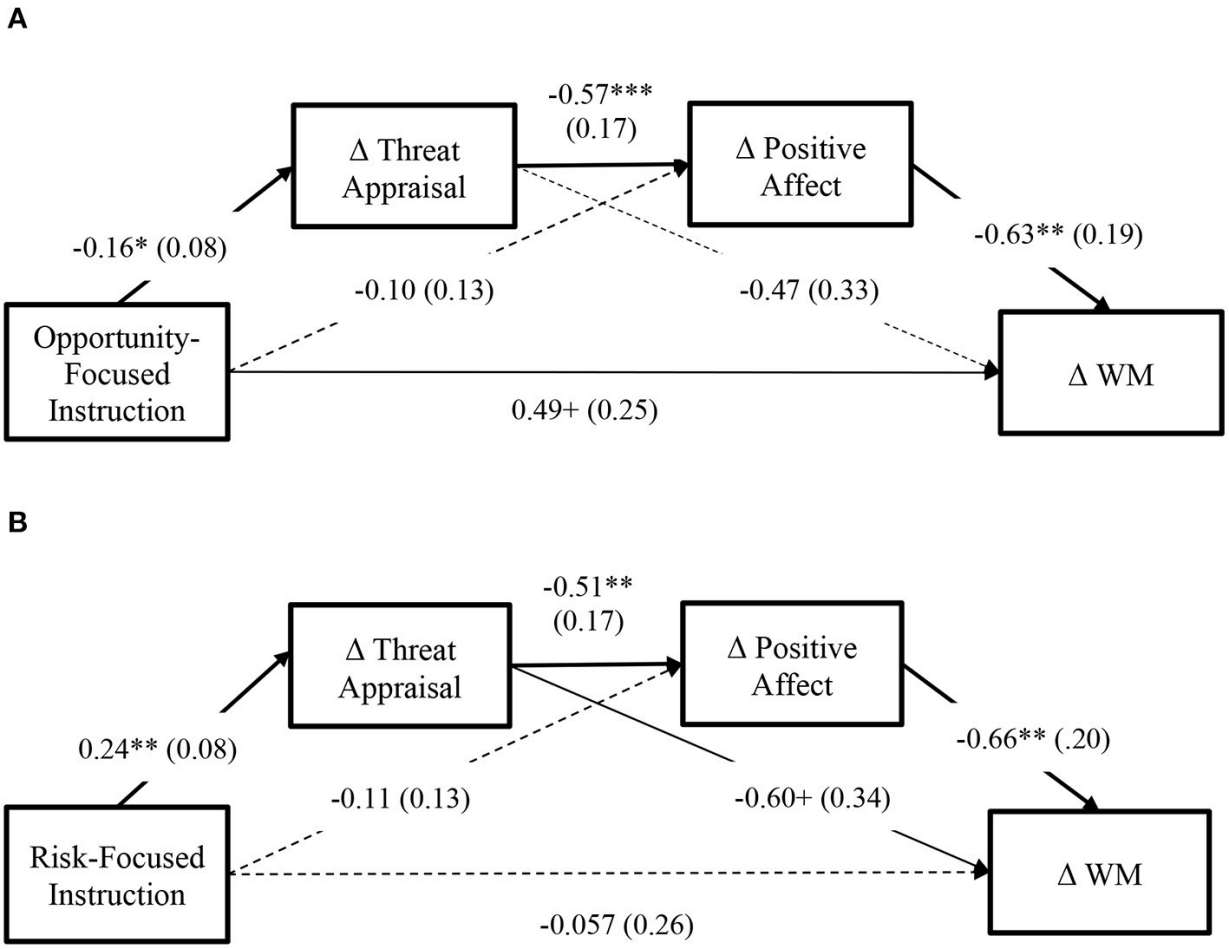
Mediation models. **(A)** Model of opportunity. **(B)** Model of risk. Coefficients and standard errors (in parentheses) are presented. Bold lines indicate statistically significant relationship. The normal solid line indicates a marginally significant path. Dashed lines indicate non-significant paths. Gender was dummy coded as 1 = girl, 0 = boy. **p < 0.01; *p < 0.05; ^+^*p* < 0.10.

Opportunity-focused task framing had a marginally significant direct effect on the improvement of WM performance, and this relationship was mediated by the alteration of threat appraisal, with the alteration of positive affect acting as the secondary mediator {indirect effect: *b* = −0.059, *SE* = 0.49, 95% *CI* [−0.22, −0.006]}. To be specific, framing the WM task as an opportunity for gain (that an individual has adequate competence to approach) can minimize the increase of threat appraisal, which was then related to a decrease of positive affect, and finally, WM performance was improved through these processes. These factors explained 14.9% of the variance in the change in WM performance. Path coefficients are presented in Figure 5A.

Risk-focused task framing adversely impacted WM performance by increasing threat appraisal {indirect effect: *b* = −0.14, *SE* = 0.093, 95% *CI* [−0.38, −0.003]}, and moreover, the increase of threat appraisal led to a decrease of positive affect, and consequently WM performance was debilitated {indirect effect: *b* = 0.081, *SE* = 0.052, 95% *CI* [0.017, 0.24]}. These factors explained 11.4% of the variance in the change in WM performance. Path coefficients are presented in Figure 5B.

To sum up, consistent with our hypothesis, the alteration of threat appraisal and that of positive affect mediated the effect of task framing on students' improvement of WM scores.

### Gender Differences

Whilst male and female students had comparable scores in all the baseline measures (*ps* > 0.10), gender difference was found in the change from pre- to post-test in threat appraisal [*F*(1, 96) = 4.24, *p* = 0.042, η^2^ = 0.045], positive affect [*F*(1, 96) = 4.71, *p* = 0.033, η^2^ = 0.049], and WM [*F*(1,96) = 6.44, *p* = 0.013, η^2^ = 0.066], but not in the alteration of challenge appraisal or negative affect (*ps* > 0.10). Importantly, we observed significant interaction effects of gender × condition on the change from pre- to post-test in threat appraisal [*F*(2, 96) = 5.54, *p* = 0.005, η^2^ = 0.11], positive affect [*F*(2, 96) = 4.27, *p* = 0.017, η^2^ = 0.086], negative affect [*F*(2, 96) = 3.90, *p* = 0.024, η^2^ = 0.011], and WM [*F*(2, 96) = 5.72, *p* = 0.005, η^2^ = 0.11], as well as a marginally significant interaction effect on the change in challenge appraisal [*F*(2, 96) = 2.90, *p* = 0.060, η^2^ = 0.060). In the risk condition, male students did not show changes from pre- to post-framing scores in any measures (*ps* > 0.10), whilst female students significantly increased their threat appraisal [*t*(17) = 5.33, *p* < 0.001] and negative affect [*t*(17) = 4.81, *p* < 0.001], decreased positive affect [*t*(17) = −2.89, *p* = 0.010], and marginally decreased WM scores [*t*(17) = 1.92, *p* = 0.072]. In the Opportunity condition, female students significantly improved their WM scores after receiving opportunity-focused task instruction [*t*(16) = 2.52, *p* = 0.023], with other states being maintained at the same levels as baselines. Results indicated that females were influenced by task instructions to a greater extent than males.

Lastly, gender was entered as the moderator into the two mediation models. We found that gender moderated the direct effect of opportunity-focused task framing on the change in WM scores {indirect effect: *b* = 1.44, *SE* = 0.49, *t* = 2.94, *p* = 0.004, 95% *CI* [0.47, 2.40]}, with the direct effect being only significant among female students {*b* = 1.09, *SE* = 0.31, *t* = 3.49, *p* < 0.001, 95% *CI* [0.47, 1.70]}, but not among male students {*b* = −0.35, *SE* = 0.38, *t* = −0.92, *p* = 0.36, 95% *CI* [−1.10, 0.40]}. Gender did not moderate other paths.

## Discussion

The present study demonstrated that task instructions concerning the potential consequences, personal competence, and goals, can influence university students' WM performance, by altering their threat appraisal and positive affect. Risk-focused task instructions debilitated WM performance through intensified threat appraisal and decreased positive affect, whereas opportunity-focused task framing can improve WM performance by minimizing the increase of threat appraisal and the decrease of positive affect. The effects of task framing were larger on females than on males.

### Task Framing Influences WM Performance

Consistent with our hypothesis, framing a WM task as an opportunity for gain, supplemented by positive feedback on personal competence to meet the task demand and the encouragement of an approach goal, significantly improved students' WM performance in our sample. In contrast, framing the task as a risk of punishment, accompanied by negative feedback on personal competence to meet the demands and the encouragement of an avoidant goal, intensified one's threat appraisal, reduced positive affect, and hindered the improvement of WM over repeated administrations.

These findings aligned with previous ones in other motivated performance situations (such as motor tasks and sports competitions): participants improved task performance after receiving information about the reward for top performers and feedback on adequate capabilities of meeting the challenge, and they outperformed those who were informed of potential punishment, high task difficulty and/or high required effort (e.g., Blascovich et al., 2004; Jones et al., 2009; Moore et al., 2012, 2013b). An approach goal was also suggested by previous research to be associated with higher levels of challenge appraisal, positive affect, and more adaptive cognitive outcomes than an avoidance goal (Adie et al., 2008; Schneider, 2008). The current research has extended the prior findings from other motivated performance contexts to WM performance. Given that WM is an importance cognitive capacity involved in higher-order cognitive processes, our findings can motivate future studies to further investigate whether the change in WM accounts for the influences of task framing on performance in other motivated performance tasks such as examinations, cognitive tasks, and social evaluation tasks.

### Task Framing Influences Appraisal and Affect

Notably, only threat appraisal but not challenge appraisal was modified by task framing in the current experiment. A threat state biases attentional processes toward negative cues, whilst a challenge state can direct attention to positive cues in an approach manner (e.g., Blascovich et al., 2004; Jamieson et al., 2012). According to Beck and Clark's (1997) Information Processing Model of Anxiety, after registering incoming information, initial threat impression and automatic negative thoughts occur automatically as a reactive process during the first stage. An individual's attention can be directed to the potential danger very rapidly, “for the survival of the organism.” During the second stage of information processing, people evaluate the situational demands and their resources in a more reflective way, after which may modify their interpretation of the specific encounter. The classic theories of appraisals (Lazarus and Folkman, 1984; Lazarus, 1991) also posited that individuals' appraisal of their own competence and resources can alter their initial impression of the situation as challenging or threatening. Hence, even very brief threatening information about the risk of loss or cost, accompanied by negative feedback on personal competence and an encouragement of an avoidance goal, may reinforce participants' interpretation of the task as a threat that they are not able to overcome.

Brief opportunity-focused task framing, however, did not successfully alter participants' cognitive appraisals. This result might be explained by positive-negative asymmetry which reflects a behavioral-adaptive mechanism (Peeters, 1971; Peeters and Czapinski, 1990). On the one hand, in order to avoid irreversible negative consequences or dangers during the interactions with environment, people's evaluations and decision making are impacted more greatly by negative stimuli than by positive stimuli of equally intense, and this impact is termed as “informational negativity effect.” During information integration and the formation of overall evaluations, negative information is more heavily weighted than positive information (Anderson, 1981). Greater weights are also accorded to potential loss or cost than to potential gain during decision making (Kahneman and Tversky, 1979, 1984). On the other hand, when the negative information is very weak, people may direct their attention toward positive stimulus and form positive hypotheses about reality (termed as “positivity bias”), in order to approach scarce potentials of positive opportunities and benefits (Peeters and Czapinski, 1990). Informational negativity effect is adaptive for people's survival, and positivity bias is adaptive for self-actualization and positive outcomes. This approach-avoidance evaluative dimension may be relevant to the occurrence of challenge and threat appraisals. The greater impact of negative stimuli than positive stimuli can explain the larger impacts of risk-focused task instruction than opportunity-focused instruction on one's cognitive appraisals. Threat appraisal can be intensified rapidly by negative information of potential loss or cost in order to avoid negative consequences, whereas challenge appraisal may require relatively more effort in directing attentional processes toward positive cues, in order to approach positive outcomes.

Similarly, the events or thoughts of a negative nature have a greater effect on one's affective responses than positive events or thoughts (Lewicka et al., 1992). The affective negativity bias can explain why opportunity-focused task framing failed to alter participants' affective responses, whilst risk-focused task framing successfully decreased positive affect in the whole sample (and increased negative affect among females). It is also noteworthy that, the influence of task framing on negative affect was less significant than that on positive affect, suggesting the distinction between positive and negative affect. Negative affect was measured by four items in this study, namely, “helpless,” “tense,” “nervous,” and “upset,” with greater intense of negativity than the opposites of positive affect measured by “happy,” “cheerful,” “energetic” and “inspired” (i.e., not happy, not cheerful, not energetic, and not inspired). Reducing positive affect does not necessarily increase negative affect. Social desirability in self-reporting lower levels of negative affect could be another reason for the non-significant effect of task framing on negative affect.

### Appraisal and Affect Mediate the Influence of Task Framing on WM Performance

More importantly, the present experiment revealed that the alteration of threat appraisal and the modification of positive affect acted as the mechanisms underlying how task framing changed students' WM performance.

The negative impact of threat appraisal on performance has been found in other cognitive tasks such as Stroop, counting backwards and complex laboratory tasks (Schneider, 2004; Moore et al., 2012; Turner et al., 2012). For example, threat appraisal has been associated with less effective attention and cognitive control (Schneider, 2004; Turner et al., 2012), fewer effective task-movements and decreased positive affect (Moore et al., 2012, 2013b), as well as more avoidance-oriented responses such as behavioral and emotional disengagement (Chen and Qu, 2021), all of which can adversely impact one's WM performance. Data supported our hypothesis about the debilitating effect of threat appraisal on WM performance. Furthermore, consistent with the classic theories of appraisals (Lazarus et al., 1980; Lazarus, 1991) and previous work (Cerin, 2003; Bryant et al., 2007; Giacobbi et al., 2007; Adie et al., 2008; Ellis et al., 2009), we found that the increase of threat appraisal resulted in a decrease of positive affect, which further led to poorer WM performance. The facilitating role of positive affect on WM has been well-discussed in previous studies (e.g., Yang et al., 2013).

The hypothesized enhancing effect of challenge appraisal on WM performance, however, could not be examined in the current experiment, because challenge appraisal was not successfully modified by brief task framing in the first place. Theoretically speaking, a challenge state may improve WM performance, given its relation to effective attention on task-relevant cues, the control of reactions to task-irrelevant cues, and positive affect in motor tasks and cognitive tasks (Blascovich et al., 2004; Jones et al., 2009; Moore et al., 2012; Turner et al., 2012), as well as approach-oriented responses such as actions taking and active coping (Chen and Qu, 2021). Future studies will benefit from using other priming or intervention methods to evoke challenge appraisal so as to examine its facilitating effect on WM performance.

### Gender Differences in the Alteration of Appraisal, Affect and WM Performance

It is interesting that the enhancing effect of opportunity-focused framing and the debilitating effect of risk-focused framing on WM performance were larger among females than males, by modifying females' threat appraisal and affective responses to a greater extent. Participants showed no gender differences in baseline WM performance, affect, and cognitive appraisals of an upcoming task with ambiguous nature and consequences. However, when “threatening” information was provided, female students rapidly increased their threat appraisal and negative affect, and decreased positive affect, which then debilitated their WM performance. Females greater attentional bias toward negative cues have been discussed in the literature (Hankin and Abramson, 2001). Females were more likely to engage in threat appraisal, avoidance-oriented responses, and negative emotional states (e.g., Chen and Qu, 2021). The current experiment took a further step to establish the relation of negative interpretational bias to poorer WM performance (directly or indirectly through reduced positive affect). Male students, did not change their cognitive appraisals or emotional responses even when confronting “potential risk of loss” in our study, possibly due to their better cognitive control of emotion than females (Koch et al., 2007). As a result, they maintained their WM performance under pressure.

The facilitating effect of opportunity-focused task framing on WM performance was only found in females. Despite the gender differences in the modification of cognitive appraisals, affect and WM performance from pre- to post-test, the relationships among these processes did not vary by gender. Our finding suggested that females might be more sensitive to new incoming information during a motivated performance task. As such, they may benefit more from intervention programs or instructional methods that aim at altering interpretational bias and improving test performance.

### Implications, Limitations, and Future Directions

The present study has made some theoretical and practical contributions to the field. Findings have enhanced our understanding of the mechanisms by which opportunity- and risk-focused task framing influence WM performance among university students. This study has not only supported the existing theories on the crucial role of cognitive appraisal in task performance and affective experiences, but also extended the research findings from other contexts to WM. Moreover, our findings can motivate future research to explore the potential cognitive mechanisms underlying the effects of task framing on other task performance (e.g., the alteration of WM may contribute to the change in performance in other complex cognitive tasks, academic examinations, and social evaluation tasks). Practically, this study has provided implications for instructional behaviors in the educational context. Opportunity-oriented instruction is recommended by the present study, to improve students' performance in motivated performance tasks such as examinations, public speaking and competitions. It is important to highlight the potential benefits and opportunities for gain instead of potential loss or harms, to encourage an approach goal rather than an avoidant goal, and to provide positive feedback on students' personal resources instead of emphasizing their inadequate competence. In addition, gender differences in the modification of cognitive appraisals, affect, and WM performance by task framing have added to the literature on affective science. Together, our findings can inform future intervention programs to modify students' interpretational or attentional biases, so as to optimize their performance under pressure.

There are also several limitations that should be addressed in future studies. Firstly, no measurement was included to evaluate whether each component (i.e., consequence, goal, self-competence) involved in the task framing was manipulated successfully. For example, we did not measure whether the “bonus” (i.e., one additional research credit) and “punishment” (i.e., additional interview) mentioned in the task instruction were really perceived by participants as gain and loss, respectively, whether they adopted an approach or avoidance goal, or whether they evaluated self-competence as adequate or inadequate after hearing the feedback. It is critical to examine the effectiveness of each component in altering cognitive appraisals, affect and WM. Secondly, we only focused on the approach-avoidance distinction of achievement goals, and did not manipulate the mastery-performance dimension. The 2 × 2 achievement goal model (Elliot and McGregor, 2001) suggested an interaction effect between approach-avoidance and mastery-performance on cognitive appraisals, affective experiences and task performance (e.g., Elliot, 2005; Adie et al., 2008). Hence, future research will benefit from manipulating and measuring both mastery-performance and approach-avoidance dimensions of achievement goals. Thirdly, most of the data were self-reported. Researchers argue that self-reported measures have limitations in assessing unconscious evaluations of a stressor (e.g., Blascovich et al., 2000; Chalabaev et al., 2009), and that self-reported psychological states are usually inconsistent with implicit measures and cardiovascular reactivity (e.g., Isen, 2008), Thus, future studies should include implicit measures and biological indicators to measure cognitive appraisals and affective experiences. Next, this study only focused on WM span, but it is indeed crucial to further examine how task framing influences accuracy and reaction time, so as to understand its influences on attentional and controlled processes. Lastly, the gender ratio was skewed in the current study with 54.8–68.6% of each group being females, due to the characteristics of our research pool. Gender differences suggested that different results might be yielded in a gender-balanced sample. Caution is required when generalizing the current findings to other samples. It is also necessary to further examine the gender effects and the underlying mechanisms using a more sophisticated experimental design in a larger sample in future studies.

## Conclusions

Task framing can influence university students' WM performance by altering their cognitive appraisals and affective experiences, with larger effects on females than on males. Framing the task as a risk of loss that a student has inadequate competence to avoid can hinder his or her improvement in WM performance, by increasing threat appraisal and decreasing positive affect. Framing a WM task as an opportunity for gain that the student has adequate competence to approach can improve WM performance, by minimizing the increase of threat appraisal and the decrease of positive affect. The current research has not only enhanced our understanding of the theoretical links among cognitive appraisals, affect and WM, but also provided practical recommendations for opportunity-oriented instruction in the educational context. Emphasizing the opportunities for gain (instead of the risk of loss), encouraging students to approach potential gain (rather than avoiding potential loss), and providing positive feedback on students' resources to meet the challenge (instead of highlighting their inadequate competence), can optimize university students' task performance, with the modification of cognitive appraisals and affective responses as the keys.

## Data Availability Statement

The raw data supporting the conclusions of this article will be made available by the authors, without undue reservation.

## Ethics Statement

The studies involving human participants were reviewed and approved by Nanyang Technological University Institutional Review Board (NTU-IRB). The patients/participants provided their written informed consent to participate in this study.

## Author Contributions

LC conceived the original idea, designed and carried out experiment, analyzed and interpreted data, and wrote the manuscript. LQ supervised the project, interpreted data, and provided critical feedback to the manuscript. Both authors contributed to the article and approved the submitted version.

## Conflict of Interest

The authors declare that the research was conducted in the absence of any commercial or financial relationships that could be construed as a potential conflict of interest.

## Publisher's Note

All claims expressed in this article are solely those of the authors and do not necessarily represent those of their affiliated organizations, or those of the publisher, the editors and the reviewers. Any product that may be evaluated in this article, or claim that may be made by its manufacturer, is not guaranteed or endorsed by the publisher.
